# Mifepristone Increases the Cytotoxicity of Uterine Natural Killer Cells by Acting as a Glucocorticoid Antagonist via ERK Activation

**DOI:** 10.1371/journal.pone.0036413

**Published:** 2012-05-01

**Authors:** Yuezhou Chen, Yan Wang, Yaling Zhuang, Feng Zhou, Lili Huang

**Affiliations:** 1 Women's Reproductive Health Laboratory of Zhejiang Province, Women's Hospital, School of Medicine, Zhejiang University, Hangzhou, Zhejiang, People's Republic of China; 2 Department of Gastroenterology, Taihe Hospital, Hubei Medical University, Shiyan, Hubei, People's Republic of China; Fudan University, China

## Abstract

**Background:**

Mifepristone (RU486), a potent antagonist of progesterone and glucocorticoids, is involved in immune regulation. Our previous studies demonstrated that mifepristone directly augments the cytotoxicity of human uterine natural killer (uNK) cells. However, the mechanism responsible for this increase in cytotoxicity is not known. Here, we explored whether the increased cytotoxicity in uNK cells produced by mifepristone is due to either anti-progesterone or anti-glucocorticoid activity, and also investigated relevant changes in the mitogen-activated protein kinase (MAPK) pathway.

**Methodology/Principal Findings:**

Uterine NK cells were isolated from decidual samples and incubated with different concentrations of progesterone, cortisol, or mifepristone. The cytotoxicity and perforin expression of uNK cells were detected by mitochondrial lactate dehydrogenase-based MTS staining and flow cytometry assays, respectively. Phosphorylation of components of the MAPK signaling pathway was detected by Western blot. Cortisol attenuated uNK cell-mediated cytotoxicity in a concentration-dependent manner whereas progesterone had no effect. Mifepristone alone increased the cytotoxicity and perforin expression of uNK cells; these effects were blocked by cortisol. Furthermore, mifepristone increased the phosphorylation of ERK1/2 in a cortisol-reversible manner. Specific ERK1/2 inhibitor PD98059 or U0126 blocked cortisol- and mifepristone-induced responses in uNK cells.

**Conclusions/Significance:**

These results suggest that mifepristone acts as a glucocorticoid antagonist to augment uNK cell-mediated cytotoxicity via ERK activation, which may be caused by increased perforin expression. These observations may reveal an important mechanism by which mifepristone upregulates the cytotoxicity of uNK cells.

## Introduction

Mifepristone (RU486) is a synthetic 19-norsteroid, and a potent antagonist of progesterone and glucocorticoids. Basic research has demonstrated a variety of potential applications for mifepristone in the fields of gynecology, endocrinology, oncology, and immunology [1]–[5]. It has been used primarily as an anti-progesterone drug to produce early pregnancy termination, and as an anti-glucocorticoid drug to ameliorate the clinical manifestations of Cushing's syndrome [6]. Recently, several studies demonstrated that for the purpose of contraception, low-dose mifepristone retards endometrial development, so-called endometrial contraception [7]. Therefore, mifepristone may serve as a novel, estrogen-free, contraceptive pill with little or no change to the menstrual cycle and few adverse side effects.

In addition to its antagonistic activities, accumulating evidence suggests that mifepristone may be involved in modulation of the immune response. *In vitro*, mifepristone significantly inhibits the proliferation of lymphocytes [5], [8] and increases the cytotoxicity of natural killer (NK) cells from pregnant subjects [9]. Uterine NK (uNK) cells are the most abundant lymphocytes in endometrial and decidual tissue, characterized as brightly staining for CD56 (CD56^+^) and dimly staining for CD16 (CD16^−^). Important roles have been proposed for uNK cells in immunotolerance, regulation of trophoblast invasion, and remodeling of the spiral arteries [10]. Altered numbers of uNK cells have been associated with recurrent miscarriage, recurrent implantation failure, fetal growth restriction, and preeclampsia [11]. For terminating early pregnancy, Lu et al. [12] demonstrated that mifepristone with misoprostol might increase the expression of CD56^+^-NK cells in decidua and induce disorder of the decidual micro-environment, which might be one mechanism underlying the abortifacient properties of this drug combination. Our previous studies showed that low-dose mifepristone increases the number of CD56^+^-NK cells, and the percentages of the NK cell subset CD3^−^CD56^+^CD16^−^ in human endometrial explants [13]. Uterine NK cell-mediated cytotoxicity and perforin expression were also augmented by treatment with mifepristone [14]. The exact mechanisms responsible for immune modulation by mifepristone in uNK cells, however, are not fully understood.

Progesterone modulates local immune responses at the maternal-fetal interface [15]. Recently, classical progesterone receptor (PR) expression was demonstrated in peripheral blood NK cells. These cells mediate the reduction of interferon (IFN)-γ secretion and the proapoptotic effects of progesterone [16]. However, human uNK cells have no PR expression [17] and progesterone cannot directly affect the biological activity of human uNK cells [18]. In contrast, the glucocorticoid receptor (GR) was identified in both uterine and peripheral blood NK cells [17], [19]. Quenby's study [20] showed that 29 women with a history of recurrent miscarriage had elevated levels of uNK cells. Oral prednisolone suppressed uNK cells from 14 to 9% between the first experimental biopsy before which the drug had not been administered and the second in which it had. These results indicated that glucocorticoid therapy has potential applications in treatment of female reproductive disorders.

In light of their immunosuppressant activity, it has been suggested that glucocorticoids might assist in preventing immune rejection of the implanting embryo [21]. These results raise the possibility that the GR is involved in the regulation of uNK cell function. Therefore, the aim of the present study was to investigate whether mifepristone acts as a glucocorticoid or progesterone antagonist to modulate the cytotoxicity of uNK cells. We also studied the role of the mitogen-activated protein kinase (MAPK) pathway as a potential mechanism underlying the effects of mifepristone on human uNK cells.

## Materials and Methods

### Tissue collection

The study was approved by the Ethics Committee of Women's Hospital, School of Medicine, Zhejiang University, China. Informed consent was obtained from all patients before tissue collection. Decidual tissue was obtained from 25 women aged 21–36 years (gestational age 5–7 weeks) who had undergone first-trimester surgical termination of pregnancy. All subjects had an ultrasound scan to confirm a viable intrauterine pregnancy and gestational age. None of them had received any hormone or immunosuppressant treatment for the previous three months.

### Preparation of uNK cells

Decidual cells were isolated from tissue as previously described [22]. Briefly, decidual samples were finely minced into approximately 1–3 mm^3^-size pieces and digested with collagenase type I (0.1%, Gibco BRL, Gaithersburg, MD, USA) and DNase I (0.01%, Applichem, Darmstadt, Germany). The cellular supernatant was consecutively filtered through 100- and 40-µm mesh screens. The cells that passed through the mesh screens were collected, resuspended and plated in phenol red-free RPMI-1640 medium supplemented with 10% charcoal-stripped fetal bovine serum (FBS; Gibco BRL), 100 IU/mL penicillin (Sigma, St. Louis, MO, USA), 100 µg/mL streptomycin (Sigma) and 1% L-glutamine (Gibco BRL). The supernatant containing decidual lymphocytes was collected. Decidual mononuclear cells were isolated by density-gradient centrifugation using Ficoll-Paque PLUS (Shanghai Chemical Reagent Factory, Shanghai, China) according to the manufacturer's instructions. Mononuclear cells were recovered from the interface and washed twice in phosphate-buffered saline (PBS). Uterine NK cells were purified with a human NK cell isolation kit (Miltenyi Biotec, Camberley, UK) and an AutoMACS instrument (Miltenyi Biotec). Cell viability was consistently ≥95% as tested by trypan blue exclusion. Isolated cells underwent suspension culture in phenol red-free RPMI-1640 medium (Gibco BRL) containing 10% charcoal-stripped FBS (Gibco BRL), 100 IU/mL penicillin (Sigma), 100 µg/mL streptomycin (Sigma), 1% L-glutamine (Gibco BRL), and 200 IU/mL recombinant human interleukin (IL)-2 (Primegen Bio-Tech, Shanghai, China).

### Flow cytometry

To confirm the purity of isolated uNK cells, the cells were washed with PBS and incubated with phycoerythrin (PE)-cyanine 5 (Cy5), PE, and fluorescein isothiocyanate (FITC)-conjugated monoclonal antibodies against CD3, CD56, CD16 or the relevant isotype control (eBioscience, San Diego, CA, USA) in the dark at 4°C for 30 min. For intracellular perforin analysis, the cells were collected, fixed with 2% paraformaldehyde, permeabilized with 0.1% Triton X-100, and then stained intracellularly with FITC-conjugated antibodies for perforin or the isotype control (eBioscience). Analyses were then conducted on a Beckman-Coulter Epics Altra Flow cytometer (Beckman-Coulter, Fullerton, CA, USA). Purity of isolated cells was confirmed to be >95% in each experiment, as shown previously [22].

### Cytotoxicity assay

Uterine NK cell-mediated cytotoxicity was evaluated with the MTS assay using the NK cell-sensitive cell line K562 (American Type Culture Collection, Manassas, VA, USA) as the target [23], [24]. The purified uNK cells were cultured in culture medium for 24 h in a 96-well U-bottom plate (BD Biosciences, Franklin Lakes, NJ, USA) in the absence or presence of different concentrations of progesterone (0.01, 0.10, 1, and 10 µM; Sigma-Aldrich), cortisol (0.01, 0.10, 1, and 10 µM; Sigma-Aldrich), or mifepristone (0.065, 0.2, and 1 µM; Sigma-Aldrich).

To investigate whether mifepristone affects uNK cell-mediated cytotoxicity by acting as a glucocorticoid antagonist, we incubated purified uNK cells with or without 1.0 µM cortisol in the presence or absence of 1.0 µM mifepristone for 24 h. Cells were then incubated with K562 cells for 4 h at an effector∶target cell ratio of 10∶1. Following co-culture, the viability of the target K562 cells was determined by the MTS assay according to the manufacturer's protocol. The percentage of cytolysis was calculated as: [1−(experimental group OD−effector cell OD)/target cell OD]×100% [25], [26], where OD is the optical density.

### Western blot

Because NK cells usually die within 24 h when cultured in vitro, uNK cells were isolated and cultured in the medium without IL-2 for 12 h as a negative control for MAPK/ERK activation. IL-15, known as a strong stimulator of NK cells, was added and used as a positive control for MAPK/ERK activation [27]–[29]. Uterine NK cells were lysed using protein lysis buffer (2% sodium dodecyl sulfate [SDS], 20% glycerol, 60 mM Tris-HCl) containing protease inhibitor. The cell lysates were centrifuged at 10,000×*g* for 10 min to remove cell debris. The supernatants were collected and denatured at 95°C for 10 min in 1× SDS loading buffer. Protein samples were diluted in 6× loading sample buffer (50 mM Tris-HCl, 100 mM dithiothreitol, 2% SDS [w/v], 10% glycerol [v/v] and a trace mount of bromophenol), resolved using 10% SDS-PAGE, and then transferred onto nitrocellulose membranes (Amersham Bioscience, Piscataway, NJ, USA). Membranes were blocked in 5% fat-free milk for 1 h and then incubated overnight at 4°C with primary antibodies against extracellular-signal-regulated kinase (ERK), phosphorylated (p)-ERK, p38 MAPK (p38), p-p38, c-Jun N-terminal kinase (JNK), and p-JNK (Cell Signaling, Danvers, MA, USA). The following day, membranes were washed (×3, for 10 min each) in PBS containing 0.1% Tween 20 and then incubated for 1 h with the corresponding secondary antibodies at room temperature. Proteins were detected with an enhanced chemiluminescence reagent (Amersham Bioscience). Density of the protein bands was measured using Quantity One software (Bio-Rad, Hercules, CA, USA).

### Data analysis

All results were expressed as means ± SEM. Before statistical analysis, the data were tested for normal distribution by applying the one-sample Kolmogorov-Smirnov test. Homogeneity of variances was evaluated by Levene's test. Statistical comparisons were performed by one-way ANOVA followed by a least significant difference test. A *P*-value<0.05 was considered significant. Statistical analyses were performed using SPSS software, version 16.0 (SPSS, Chicago, IL, USA).

## Results

### Cortisol increases uNK cell-mediated cytotoxicity but not progesterone

**Figure 1:**
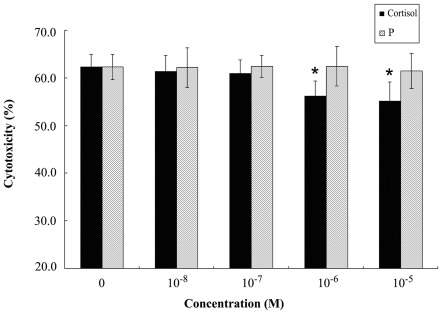
Analysis of uNK cell-mediated cytotoxicity to target cells (K562) using the MTS assay. Uterine NK cells were treated with different concentrations of progesterone or cortisol for 24 h, then uNK cell-mediated cytotoxicity was evaluated by the MTS assay using the NK cell-sensitive cell line K562.

Human uNK cells were isolated by immunomagnetic separation and treated with different concentrations of progesterone or cortisol. As shown in Figure 1, treatment with progesterone from 0 (control) to 10.0 µM did not change uNK cell-mediated cytotoxicity towards K562 cells. In contrast, concentrations of cortisol ≥1.0 µM caused a significant decrease in the effective cytotoxicity of uNK cells. While the uNK cell-mediated cytotoxicity without cortisol was 62.3±2.7%, extents of cytotoxicity after cortisol treatments were 56.2±3.1% and 55.1±4.0% at doses of 1 µM and 10 µM, respectively (*P*<0.05 for both).

### Mifepristone increases uNK cell-mediated cytotoxicity, an effect reversed by cortisol

**Figure 2:**
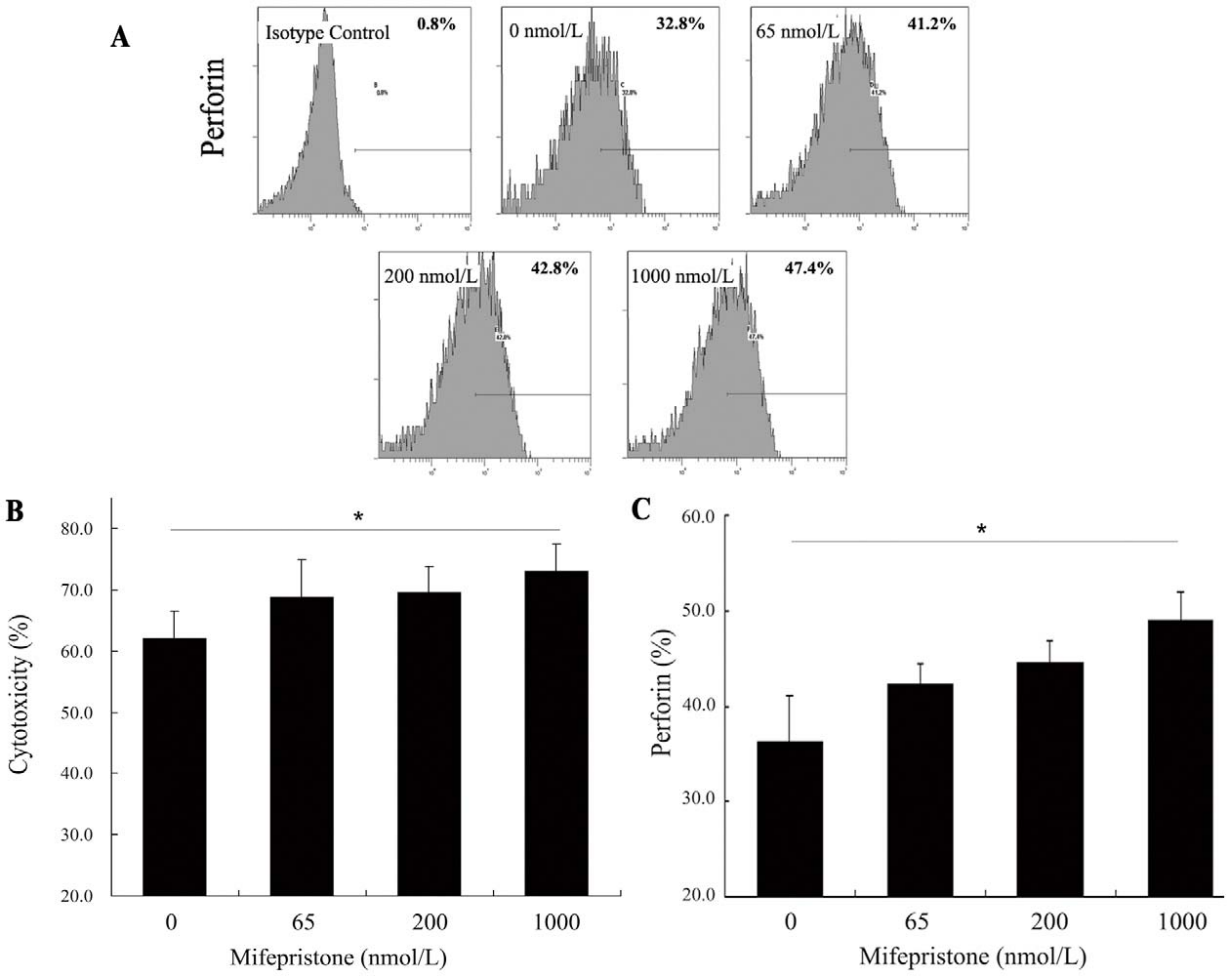
Analysis of uNK-cell cytotoxicity and perforin expression after treatment with different concentrations of mifepristone. The purified uNK cells were incubated with 0, 65, 200, and 1000 nmol/L mifepristone for 24 h. Then, they were subjected to analysis of uNK-cell cytotoxicity and perforin expression. A, a representative flow cytometry profiles of perforin expression was shown. Uterine NK-cell cytotoxicity (B) and perforin (C) expression after treatment with different concentrations of mifepristone were evaluated. Values are expressed as means ± SEM. The influence of mifepristone was evaluated by 4 independent experiments. *P-*value*s* referred to One-way analysis of variance, n = 6, * *P*<0.05 vs. control group.

It has been reported that when mifepristone was administered at a dose of 1 mg/day, endometrial development appeared to be slightly retarded and the steady plasma level of mifepristone was found to be 65 nmol/L. When the serum mifepristone concentration reached 232.7 nmol/L, ovulation was inhibited. 1000 nmol/L is the steady plasma level of mifepristone with 10 mg/day that achieved the purpose of emergency contraception [30]. We found that, 65 and 200 nmol/L mifepristone had no significant influence on human uNK-cell cytotoxicity in vitro. Compared with control group, human uNK-cell cytotoxicity (73.16±4.27% vs. 62.24±4.39%, *P*<0.05) (Fig. 2B) significantly increased in 1000 nmol/L (1.0 µM) mifepristone group.

**Figure 3:**
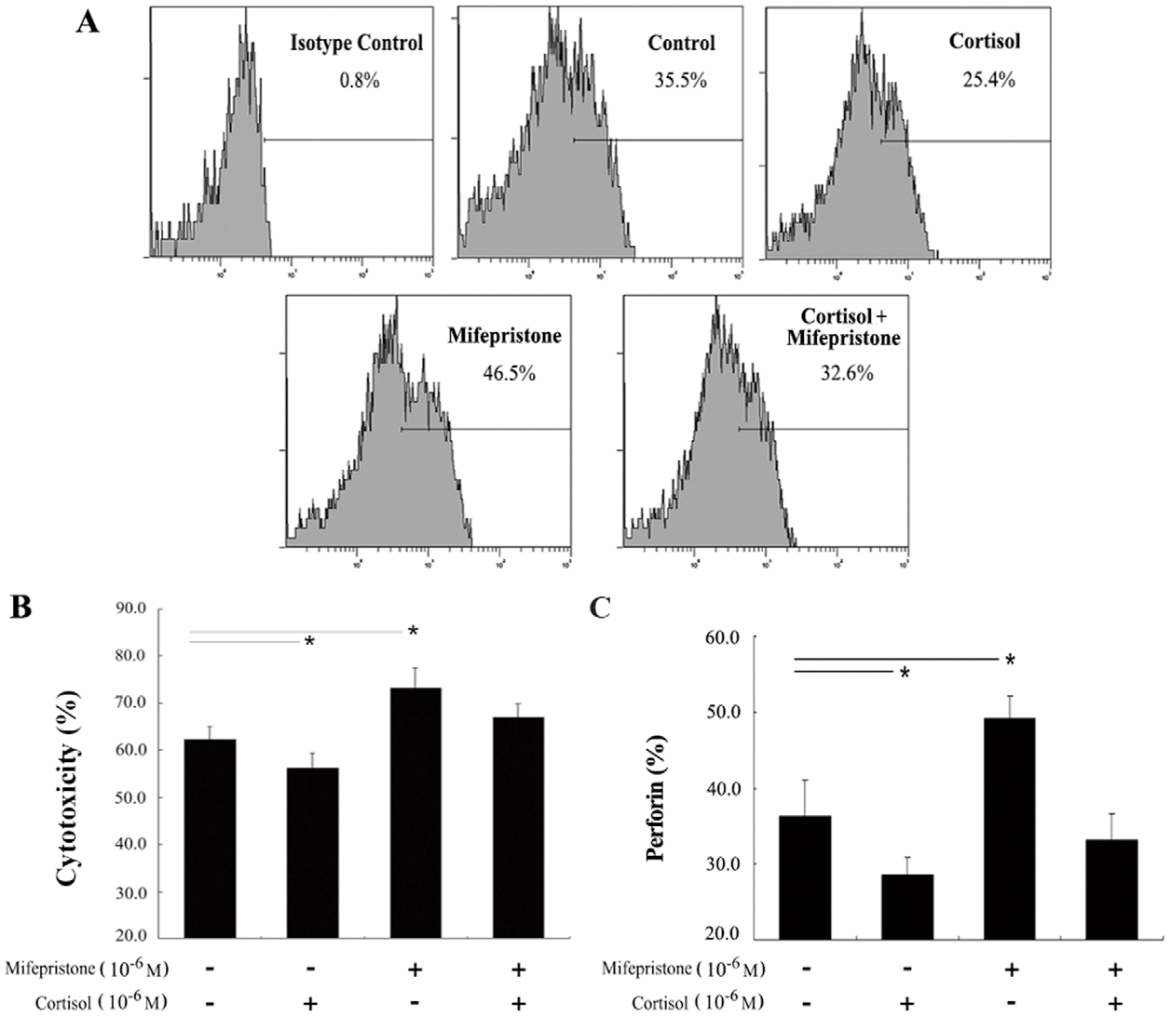
Effects of cortisol on mifepristone-induced uNK-cell cytotoxicity and perforin expression. Isolated uNK cells were treated with cortisol (1.0 µM) ± and mifepristone (1.0 µM) for 24 h. A, a representative flow cytometry result for perforin expression in different groups. B, results of uNK-cell cytotoxicity in different groups. C, data summary of flow cytometry results for perforin expression. The value is the percent of perforin-positive cells in the total number of uNK cells. Experiments were independently repeated 5 independent experiments. Data were analyzed using ANOVA and expressed as means ± SEM. *, *P*<0.05.

Uterine NK cells were then treated without or with mifepristone (1.0 µM) in the presence or absence of 1.0 µM cortisol. Mifepristone without cortisol increased uNK cell-mediated cytotoxicity (62.3±2.7% vs. 73.2±4.3%, *P*<0.05) and this effect was reversed by cortisol (73.2±4.3% vs. 66.9±2.9%, *P*<0.05; Fig. 3B).

### Upregulation of perforin expression by mifepristone in uNK cells is reversed by cortisol

We found that, 65 and 200 nmol/L mifepristone had no significant influence on human uNK-cell perforin expression in vitro. Compared with control group, human uNK-cell perforin expression (49.13±2.92% vs. 36.23±0.85%, *P*<0.05) (Fig. 2C) significantly increased in 1000 nmol/L (1.0 µM) mifepristone group.

We then explored the effects of cortisol on changes in perforin expression induced by mifepristone in uNK cells. Cortisol (1.0 µM) significantly inhibited the mifepristone-induced increase in perforin expression (36.2±4.9% vs. 28.5±2.3%, *P*<0.05) and mifepristone significantly increased perforin expression (36.2±4.9% vs. 49.1±2.9%, *P*<0.05). When uNK cells were treated with mifepristone (1.0 µM) in the presence of cortisol, the upregulation of perforin expression by mifepristone in uNK cells was suppressed (49.1±2.9% vs. 33.1±3.5%, *P*<0.05; Fig. 3C).

### Mifepristone increases MAPK/ERK activation in uNK cells

**Figure 4:**
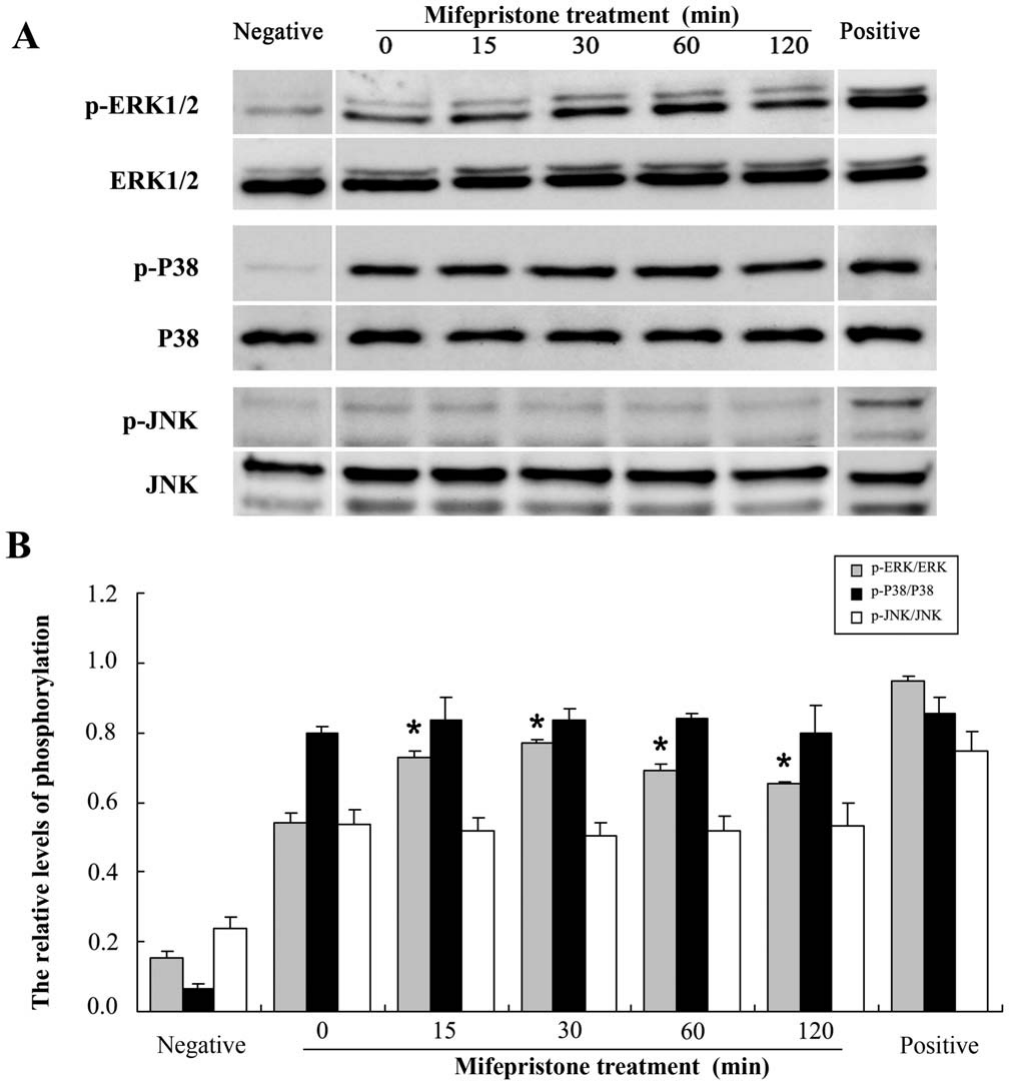
Effects of mifepristone on the phosphorylation level of MAPK in uNK cells. A, a representative immunoblot is shown after the treatment of uNK cells with mifepristone (1.0 µM) for 0, 15, 30, 60, and 120 min. Uterine NK cells were isolated and cultured in the medium without IL-2 for 12 h as a negative control of MAPK/ERK activation. In the drug treatment group, uNK cells were cultured in the medium supplemented with IL-2. Uterine NK cells were cultured in the medium with IL-2 and IL-15 for 15 min as a positive control. Immunodetection of MAPK members used specific antibodies for phosphorylated and total proteins of ERK1/2, P38 and JNK. B, densitometric scans of triplicate blots are shown. Experiments were independently repeated 3 times in each group. Data were analyzed using ANOVA and expressed as means ± SEM. *, *P*<0.05.

**Figure 5:**
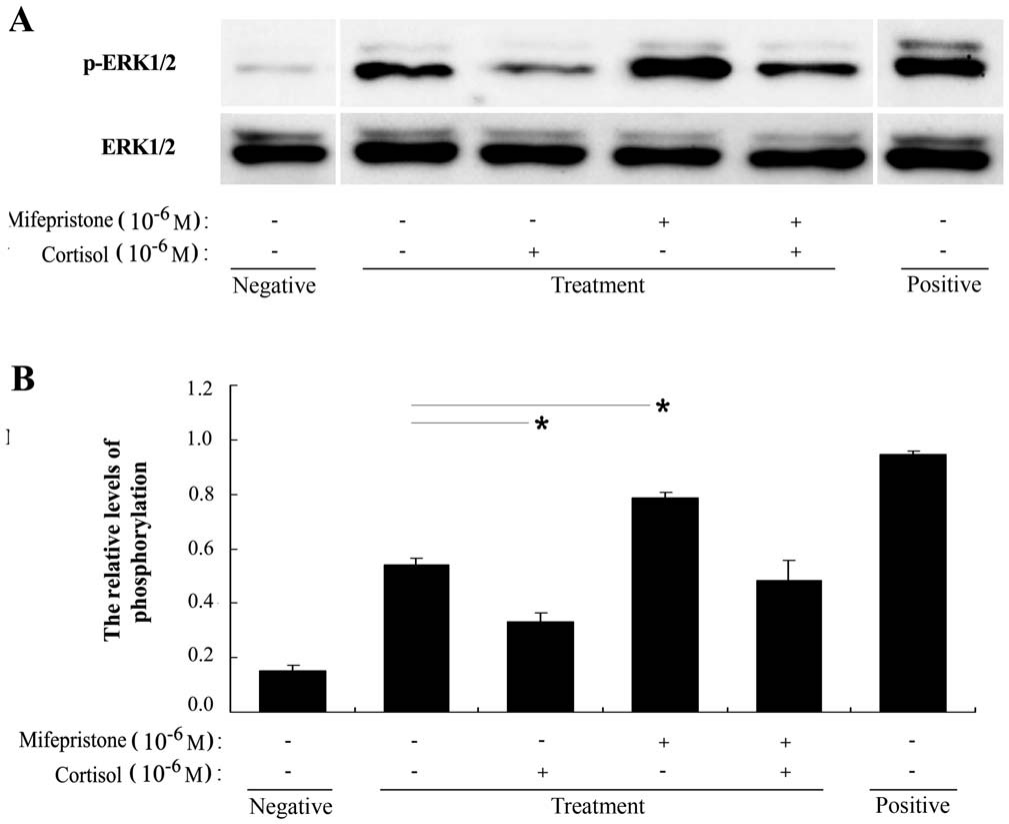
Effects of mifepristone on the ERK signaling pathway in uNK cells. Western blot of p-ERK and total ERK in uNK cells treated with mifepristone ± cortisol. To detect p-ERK and total ERK, total cell lysates were harvested after a 30 min treatment with mifepristone (1.0 µM) ± cortisol (1.0 µM). Uterine NK cells were isolated and cultured in the medium without IL-2 for 12 h as a negative control of MAPK/ERK activation. In the drug treatment groups, uNK cells were cultured in the medium supplemented with IL-2. Uterine NK cells were cultured in the medium with IL-2 and IL-15 for 15 min as a positive control. A typical blot (A) and densitometric scans of triplicate blots (B) are shown. Data were analyzed using ANOVA and expressed as means ± SEM. *, *P*<0.05.

To confirm whether or not the MAPK pathway is involved in immune regulation by mifepristone, the expression and activation of ERK, p38 and JNK in uNK cells were determined by Western blot. Uterine NK cells were treated with 1.0 µM mifepristone for different times. The results indicated that mifepristone induced a time-dependent activation of the ERK pathway. Activation of ERK in uNK cells occurred at 15 min after mifepristone stimulation, peaked at 30 min, and decreased at 60 and 120 min (Fig. 4). In contrast, the phosphorylation levels of p38 and JNK in uNK cells were not altered at the different time points after mifepristone stimulation. Furthermore, 1.0 µM mifepristone significantly increased ERK activation at 30 min after the stimulation (*P*<0.05) and 1.0 µM cortisol significantly inhibited ERK activation (*P*<0.05). When uNK cells were simultaneously treated with cortisol and mifepristone, activation of the ERK pathway by mifepristone was suppressed (*P*<0.05, Fig. 5).

### Mifepristone augments uNK cell-mediated cytotoxicity and perforin expression by activation of the ERK pathway

**Figure 6:**
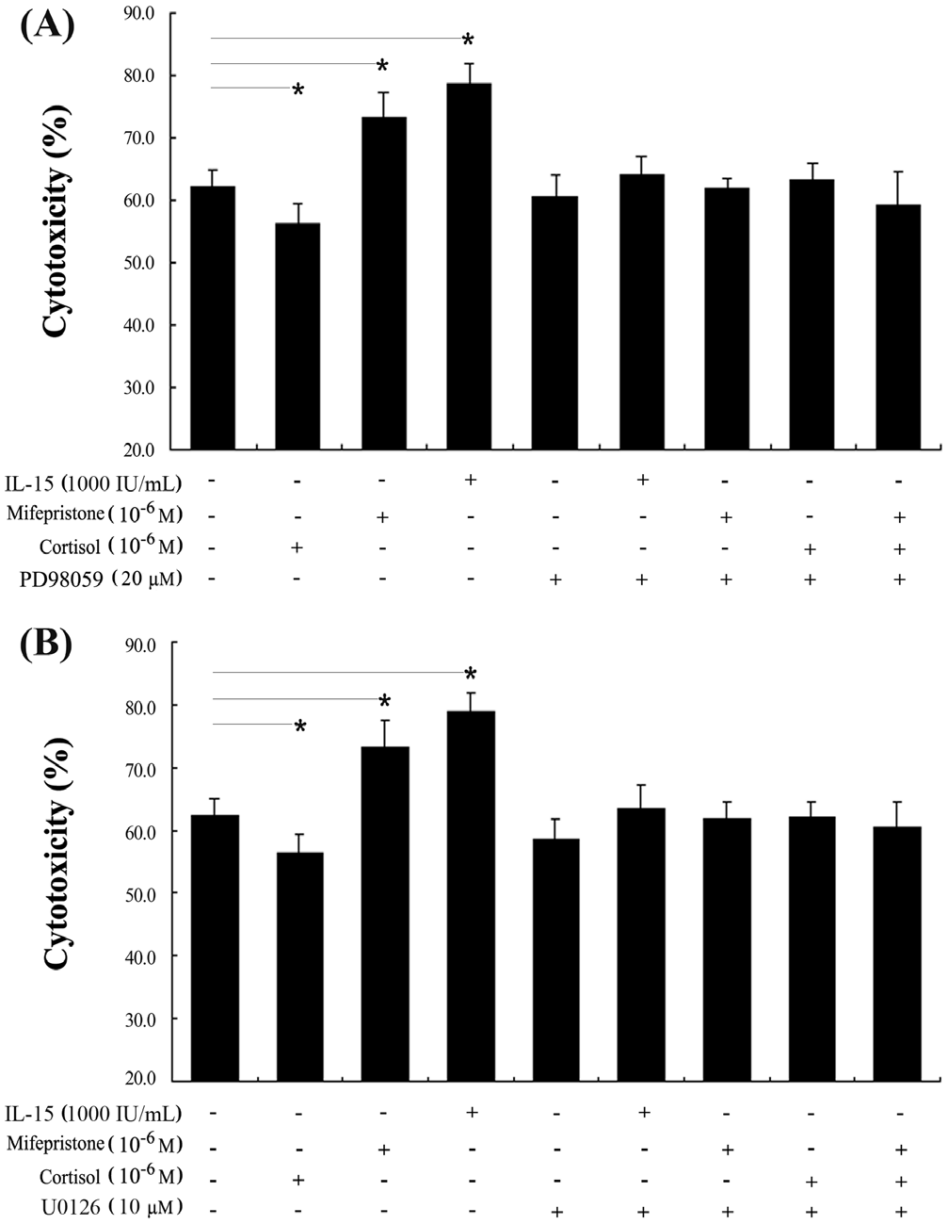
The increased cytotoxicity of uNK cells by mifepristone was inhibited by PD98059 or U0126. Before the treatment with cortisol ± mifepristone, uNK cells were pretreated with PD98059 (A) or U0126 (B) for 30 min. They were then treated with mifepristone ± cortisol for 24 h. Cells stimulated with IL-15 were used as positive control. The cytotoxicity of uNK cells was detected by the MTS assay. Data were analyzed using ANOVA and expressed as means ± SEM. *, *P*<0.05.

**Figure 7:**
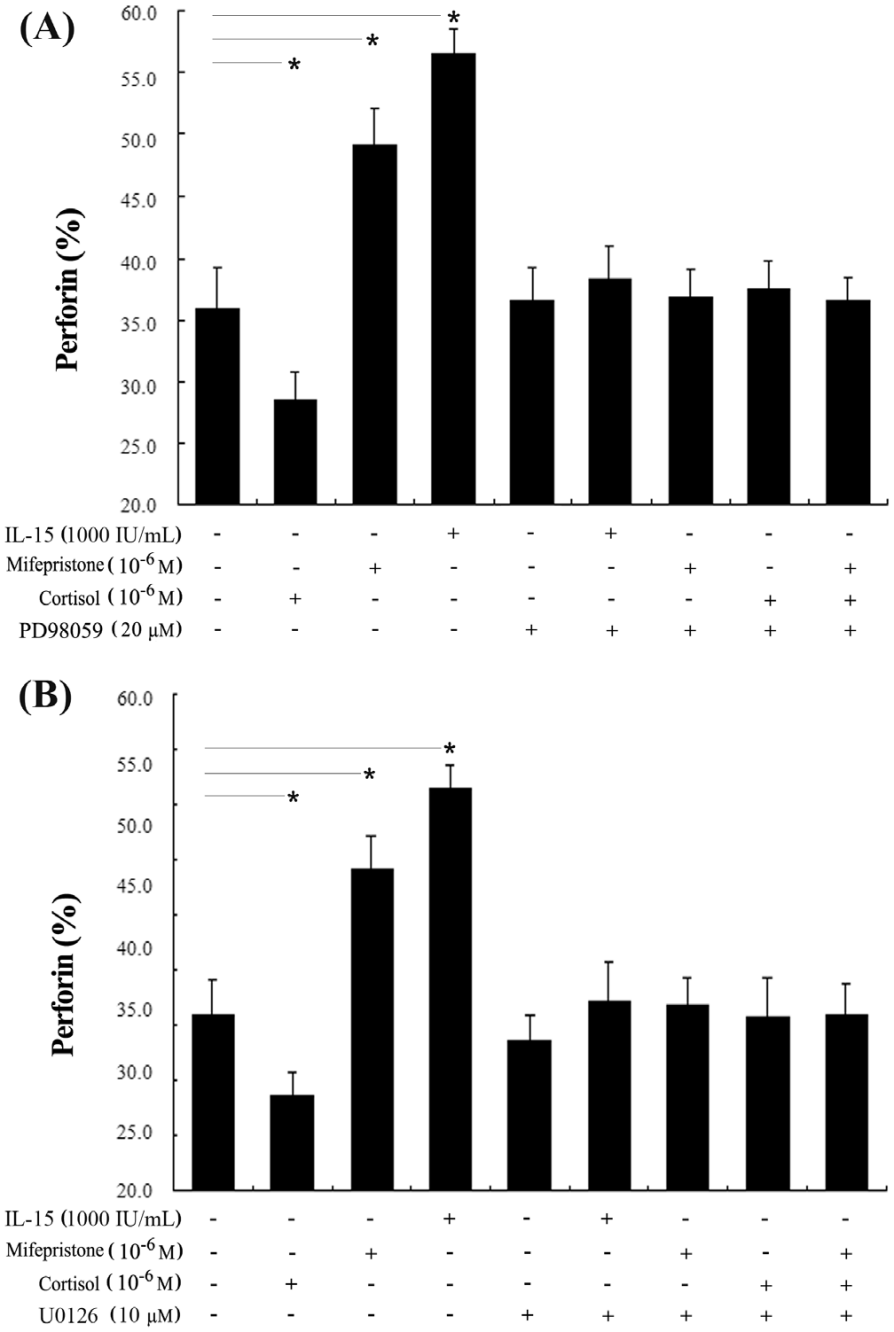
Upregulation of perforin expression by mifepristone was inhibited by PD98059 or U0126. Before the treatment with cortisol ± mifepristone, uNK cells were pretreated with PD98059 (A) or U0126 (B) for 30 min. They were then treated with mifepristone ± cortisol for 24 h. Cells stimulated with IL-15 were used as positive control. Perforin expression in uNK cells was examined by flow cytometry. The value is the percent of perforin-positive cells in the total number of uNK cells. Data were analyzed using ANOVA and expressed as means ± SEM. *, *P*<0.05.

Because mifepristone appeared to increase ERK activation, we further investigated the possible role of the ERK pathway in mifepristone-induced cytotoxicity and changes in perforin expression in uNK cells. Uterine NK cells were pretreated for 30 min with either of two specific ERK1/2 inhibitors, PD98059 or U0126. They were then treated with mifepristone with or without cortisol for 24 h. The effect of mifepristone ± cortisol on uNK cell-mediated cytotoxicity was determined by the MTS assay. After pretreatment with PD98059 or U0126, mifepristone had no effect on uNK cell-mediated cytotoxicity, with or without cortisol (*P*>0.05, Fig. 6). No changes in perforin expression, due to cortisol or mifepristone, were observed when uNK cells were pretreated with PD98059 or U0126 (Fig. 7).

## Discussion

Accumulating evidence supports a requirement for optimal postovulatory increases of uNK cell numbers for successful embryo implantation and placentation [31], [32]. Altered numbers of uNK cells and their function may be responsible for reproductive disorders [11]. Our previous research confirmed that low-dose mifepristone increases the number of CD56^+^-NK cells and the percentages of the NK cell subset CD3^−^CD56^+^ CD16^−^ in human endometrial explants [13]. Mifepristone can also directly augment uNK cell-mediated cytotoxicity through the increased expression of perforin (unpublished result). However, the specific mechanisms responsible for the effects due to mifepristone in uNK cells are unclear.

Changes in the production of steroids play a major role in modulation of local immunosuppression at the maternal-fetal interface. The number of uNK cells within the endometrial stroma varies during the menstrual cycle and throughout pregnancy. It is tempting to speculate that progesterone may be involved in the ever-changing status of uNK cells. In the present study, however, we found that progesterone at different concentrations does not affect uNK cell-mediated cytotoxicity.

Despite expression of the classical PR in peripheral blood NK cells, strong evidence indicates that neither isoform of the PR is expressed in uNK cells [17]. The proliferation, target cell lysis and cytokine secretion of high-purity uNK cells was not influenced by progesterone *in vitro* [18]. The current results are consistent with previous studies and support the concept that progesterone has no direct effect on the biological activity of uNK cells.

We found that mifepristone augments the cytotoxicity of uNK cells in culture, and this effect was blocked by cortisol but not progesterone. This suggested that mifepristone could regulate uNK cell-mediated cytotoxicity by antagonizing the GR rather than the PR. Accumulating evidence indicates that mifepristone has a variety of putative effects on cancer cells and immune cells [1]–[5], with the mechanism of action involving antagonism of hormone receptors [33], [34]. Binding of mifepristone to the receptor induces the loss of associated heat shock proteins and results in receptor dimerization. The mifepristone-receptor dimer also binds to hormone response elements and activates some genes as a partial glucocorticoid agonist if glucocorticoid is absent. When both glucocorticoids and mifepristone are present, the mifepristone-receptor dimer is transcriptionally inactive as an antagonist of glucocorticoids [35].

Mifepristone binds to the human GR with an affinity 18 times greater than that of cortisol [36]. Because mifepristone blocks the GR in a competitive manner, the effect can be reversed by glucocorticoid administration [37]. The GR, but not the PR, was identified in human uNK cells [17]. Therefore, it is reasonable that the increased cytotoxicity of uNK cells induced by mifepristone is mediated by action at the GR. In addition, the opposite effects due to mifepristone and cortisol on uNK cell-mediated cytotoxicity and perforin expression in the present study indicated that mifepristone acts as an antagonist rather than agonist.

NK cells are important for early host defense against infection and tumors [38], [39]. They exert cytotoxic effects primarily through granule exocytosis [40], [41]. Perforin is a cytolytic mediator that is released by cytoplasmic granules and promotes the death of target cells [42]. Upon release into the immunologic synapse, perforin induces lysis of target cells [43]. Through polymerization and insertion into target cells, perforin causes osmotic leakage and provides access to other toxic granules, which results in lysis and apoptosis of the target cells [44]. NK cell-mediated cytolysis is highly dependent on perforin [45]. It has been reported that the mobilization and redistribution of perforin play an important role in the control of NK cytotoxicity [46]. In a previous study, we showed that in human endometrial explants, mifepristone increased expression of perforin and increased uNK cell-mediated cytotoxicity [14]. In the current study, we observed that the mifepristone-induced increase in perforin expression in uNK cells can be reversed by cortisol, which indicated that mifepristone could increase the expression of perforin through the GR.

Glucocorticoids are known to show an immunosuppressive effect in lymphocytes. In the clinic, glucocorticoids work as immunosuppressive drugs to impair NK-cell function by affecting the expression and function of NK receptors [47]. Previous study showed that the suppression of human lymphocyte proliferation by cortisol can be reversed by mifepristone; however, the suppression by progesterone is not affected [8].

In the present study, we found that activation of the ERK pathway by mifepristone was blocked by the presence of cortisol. This suggests that mifepristone can promote activation of the ERK pathway through the GR. Moreover, we found that when ERK activation was inhibited by PD98059 or U0126, neither uNK cell-mediated cytotoxicity nor perforin expression was influenced by either cortisol or mifepristone. This implied that the activation of ERK by mifepristone was involved in the regulation of uNK cell-mediated cytotoxicity. Usually, the MAPK signaling pathway is activated through membrane receptors. Although GR is a member of the nuclear hormone receptor superfamily, it has been shown that glucocorticoids induce the expression of the glucocorticoid-leucine zipper (GILZ) and MAPK phosphatase-1 (MKP-1). The induction of GILZ and MKP-1 genes provides a mechanism by which GR can modulate the MAPK pathway [48].

NK cell function is controlled by signals generated from inhibitory and stimulatory receptors. Ligation of the stimulatory receptor leads to activation of a cascade of intracellular signaling events, resulting in exocytosis of granules to lyse target cells [49], [50]. Many receptor signaling molecules, including NKG2D (the best-characterized activating receptor of NK cells), are located upstream of the MAPK pathway and are involved in controlling perforin and granzyme B release from NK cells [46], [51]–[53]. It has also been demonstrated that both the ERK and JNK MAPK pathways are involved in NKG2D-mediated cytotoxicity [51]. In addition, glucocorticoids can trigger their biological functions through the MAPK pathway [54]–[56]. Because mifepristone is an antagonist of glucocorticoids, it was expected that the same pathway would be involved in the regulation of uNK cell-mediated cytotoxicity by mifepristone.

Our findings indicate that mifepristone may act as a glucocorticoid antagonist to augment uNK cell-mediated cytotoxicity through the ERK pathway. We suggest this as a possible mechanism by which mifepristone contributes to the increased cytotoxicity of uNK cells, which plays an important role in endometrial contraception. In conclusion, our findings indicate that mifepristone may play a pivotal role in the control of inflammatory responses and suggest a new clinical application for mifepristone.
